# A multi-stage lithium-ion battery aging dataset using various experimental design methodologies

**DOI:** 10.1038/s41597-024-03859-z

**Published:** 2024-09-19

**Authors:** Florian Stroebl, Ronny Petersohn, Barbara Schricker, Florian Schaeufl, Oliver Bohlen, Herbert Palm

**Affiliations:** 1 Institute for Sustainable Energy Systems (ISES), Munich University of Applied Sciences, Munich, 80335 Germany; 2 Hoppecke Systemtechnik GmbH, Advance Development, Zwickau, 08056 Germany; 3 Siemens AG, Technology, Erlangen, 91058 Germany

**Keywords:** Electrical and electronic engineering, Batteries, Scientific data

## Abstract

This dataset encompasses a comprehensive investigation of combined calendar and cycle aging in commercially available lithium-ion battery cells (Samsung INR21700-50E). A total of 279 cells were subjected to 71 distinct aging conditions across two stages. Stage 1 is based on a non-model-based design of experiments (DoE), including full-factorial and Latin hypercube experimental designs, to determine the degradation behavior. Stage 2 employed model-based parameter individual optimal experimental design (pi-OED) to refine specific dependencies, along with a second non-model-based approach for fair comparison of DoE methodologies. While the primary aim was to validate the benefits of optimal experimental design in lithium-ion battery aging studies, this dataset offers extensive utility for various applications. They include training of machine learning models for battery life prediction, calibrating of physics-based or (semi-)empirical models for battery performance and degradation, and numerous other investigations in battery research. Additionally, the dataset has the potential to uncover hidden dependencies and correlations in battery aging mechanisms that were not evident in previous studies, which often relied on pre-existing assumptions and limited experimental designs.

## Background & Summary

The rapid growth in the use of lithium-ion (Li-ion) batteries across various applications, from portable electronics to large scale stationary battery energy storage systems (BESS), underscores the critical importance of understanding their degradation behavior. Characterizing battery aging is crucial for improving battery performance, lifespan, and safety. Achieving this requires a dataset specific to the cell type and ideally tailored to the target application, which often involves time-consuming and expensive measurement campaigns. To address these challenges, NASA Ames Research Center^1–4^ and CALCE^5–10^ were the first to make Li-ion battery aging datasets publicly available. Subsequently, the availability of publicly accessible datasets from various scientific and industrial sources has expanded. These datasets can be broadly categorized according to their specific research objectives: investigating calendar or cyclic aging mechanisms and aging modes^1,4,11–37^, exploring the effect of cell-to-cell variations on aging^18,33,38–40^, using estimators or, more commonly, training machine learning models to predict battery capacity, lifetime and performance^2–4,6–8,10,32,41–65^, analyzing the effect of fast charging in general or (fast) charging protocols in particular on battery aging^29,30,45,46,66–68^, and investigating the aging behavior of Li-ion batteries under real-world operating conditions^32,41,42,69–77^. A more detailed overview of the above mentioned datasets is given by Dos Reis *et al*.^78^, Hassini *et al*.^79^, Mayemba *et al*.^80^ and Thelen *et al*.^65^.

Despite the importance of Li-ion aging datasets, optimization of the underlying experimental designs is only rarely applied. This could, however, significantly enhance the information content and efficiency of the experiments. Diao *et al*. utilized a full-factorial experimental design^14,81^, while Attia *et al*. applied an Optimal Experimental Design (OED) for planning fast-charging protocols after first 100 cycles^68^ and Wildfeuer *et al*. implemented a D-optimal design for their cyclic test series, but details of the methodology remain unclear^33,34^. Prior assumptions or expert knowledge were used to design the test conditions for the majority of the aforementioned datasets. Comprehensive, published datasets on the results of Li-ion battery aging measurements based on optimized experimental designs, which also allow a comparability of the experimental design methodology in terms of their quality of parameter estimation impact, are not yet available.

This study aims to overcome limitations of previous research on Li-ion battery aging by using advanced design of experiments (DoE) methods to generate a comprehensive aging dataset. The primary objective is to quantify and validate the effectiveness of optimal experimental design (OED) approaches in this context. For this purpose, a multi-stage experimental approach is used. Its first stage is based on a a non-model-based DoE (full-factorial and Latin hypercube sampling) to determine the baseline degradation behavior. Subsequently, a model-based parameter individual optimal experimental design (pi-OED) is implemented to refine specific dependencies and according model parameters based on the initial findings. In the second stage, the same non-model-based sampling design as in the first phase is supplemented to allow direct comparison and validation with the OED methodology and to quantify its impact on the quality of parameter estimation.

Encompassing a total of 279 tested cells with 71 test points across two stages, the generated dataset is not only designed to validate the benefits of optimal experimental design but also holds substantial potential for a wide range of applications. These include training machine learning models for battery life prediction, calibrating physics-based or (semi-)empirical models for battery performance and degradation, investigating the impact of various aging factors under controlled conditions, and uncovering hidden dependencies and correlations in battery aging mechanisms that were not previously evident.

Notably, the scope of reference performance tests (RPTs) was varied throughout the experiment, minimizing their influence on degradation. The inclusion of test points with and without RPTs in the calendar aging study further enables the quantification of RPTs’ impact on cell aging. The presented battery aging study presents several unique aspects and significant contributions to the field of battery research:

- **Comprehensive Experimental Design:**
  The study employs a multi-stage experimental design, including both preliminary exploration and optimized experimental design (pi-OED), providing a robust basis for understanding chances and challenges of using OED for battery aging studies.
- **Wide Range of Experimental Conditions:**
  The dataset encompasses a broad spectrum of experimental variables, including a wide range of application-related experimental conditions, focusing on temperatures, various average states of charge (SOC), charge/discharge current rates and depths of discharge (DOD), offering a holistic view of battery aging processes.
- **High Sampling Density:**
  With 279 individual cells in 93 different aging conditions, the study features high sampling density, ensuring detailed and accurate data collection that captures nuanced aging behaviors under various conditions.
- **Combination of Calendar and Cycle Aging Data:**
  By including both calendar and cycle aging data, the dataset provides a comprehensive perspective on battery degradation, supporting diverse research needs and applications.
- **RPTs with Varying Scopes:**
  The study includes reference performance tests (RPTs) with varying scopes to reduce their impact on degradation.
- **Transparency and Accessibility:**
  All data files are available under a CC-BY-4.0 license at Figshare, promoting transparency and facilitating further research and collaboration within the scientific community.

These aspects collectively contribute to advancing the understanding of lithium-ion battery aging and provide valuable insights for future research and development in battery technology.

## Methods

The presented study involved experimental characteriziation of Li-ion battery aging under common influence factors, i.e. ambient temperature (*T*_amb_), maximum state of charge ($SO{C}_{\max }$), depth of discharge (*DOD*), and charge and discharge current/C-rate (*C*_ch_, *C*_dch_).^82–85^

### Experimental setup

For this study, the commercially available Samsung INR21700-50E with a rated capacity of 4.9 Ah, a maximum charge current of 4.9 A / 1 C, and a maximum discharge current of 9.8 A / 2 C was used^86^. The study was conducted across three laboratories: Siemens (SIE), Intilion (INT), and the University of Applied Sciences Munich (HM). (During the project, the involved division at *Intilion* transitioned to *Hoppecke*. However, INT was used as the lab identifier for the duration of the study.) Each lab followed a standardized setup and used the same test protocol to ensure consistency in the results.

**Fig. 1:**
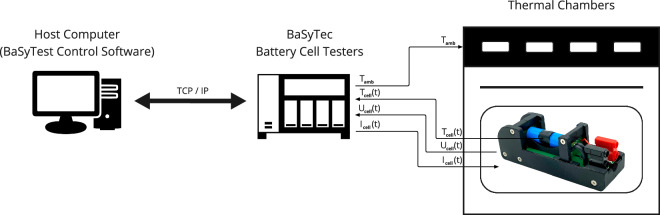
General test setup of this study. The setup comprises a host computer running BaSyTest Control Software, connected via TCP/IP to multiple BaSyTec battery cell testers. These testers are interfaced with thermal chambers to control ambient temperature (*T*_amb_). The system measures and controls current (*I*_*cell*_(*t*)), voltage (*U*_*cell*_(*t*)), and temperature (*T*_*cell*_(*t*)) of each battery cell during testing. This configuration is established at all involved laboratories.

The experimental setup, as illustrated in Fig. 1, includes multiple battery cells inside a temperature chamber with controlled ambient temperature (*T*_amb_, *amb_temp*). In all labs, cells are connected to the BaSyTec cell testing devices specified below to measure cell voltage (*U*(*t*), *c_vol*), cell current (*I*(*t*), *c_cur*) and cell surface temperature (*T*_cell_(*t*), *c*_*surf*_*temp*) according to the same test protocol. The BaSyTec testers itself are connected to host computers running the BaSyTest Control Software (V6.2.47 (stage 1), V6.2.53 (stage 2)) for tester management and data export.

The specific configurations for each laboratory are as follows (with production year in brackets):

- **Siemens (SIE)**: Cell testers: One BaSyTec GSM (2009) tester and one BaSyTec XCTS (2012) tester; Temperature chambers: one Binder KB53 (2003) and one Binder KB115 (2020).
- **Intilion (INT)**: Cell testers: Two BaSyTec CTS Lab (2009) testers and one BaSyTec GSM (2009) tester; Temperature chambers: two Binder MK240 (2011).
- **Munich University of Applied Sciences (HM)**: Cell testers: Two BaSyTec CTS (2021) testers; Temperature chambers: two ATT DY200T (2021), one Binder KT53 (2022) and one Binder BD56 (2021).

In all setups, individual battery cells were fixed in custom cell holders throughout the study. The cell holders use three Feinmetall pins (F713(C)) to contact the cells, and enable four-wire voltage measurements for accurate readings. Temperature sensors (Epcos B57861S0502 NTC 5k) were placed directly onto the cell surface and were thermally shielded to ambience by foam (approx. 9 × 9 mm^2^) and isolation tape. Cycle aging cells were connected to testers during RPTs and cycle aging phase, whereas calendar aging cells were only connected during RPTs, and disconnected during calendar aging intervals to imitate open-loop storage.

**Fig. 2:**
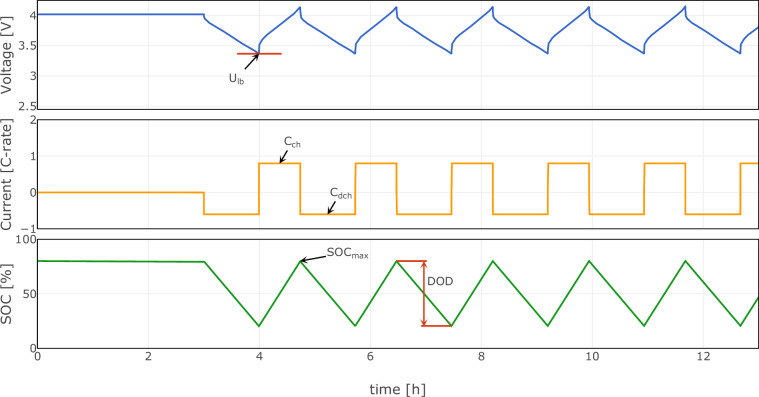
Aging protocol used in this study. This figure illustrates the aging protocol used for characterizing the degradation of lithium-ion batteries, highlighting the experimental degrees of freedom including maximum state of charge ($SO{C}_{\max }$), depth of discharge (*DOD*), charge current (*C*_ch_), and discharge current (*C*_dch_). The protocol establishes a lower voltage bound (*U*_lb_) that is fixed after the initial discharge until the specified depth of discharge is reached. In subsequent cycles, *U*_lb_ serves as the limit for discharge, while *DOD* becomes the termination criterion during the charging process to avoid SOC drift due to current integration errors. The graph plots voltage, current (C-rate), and state of charge over time, demonstrating the cyclical nature of the testing protocol after an initial 3 h tempering pause at *T*_amb_.

The aging profile designed to assess the impact of temperature (*T*), state of charge (*SOC*), depth of discharge (*DOD*), charge rate (*C*_ch_), and discharge rate (*C*_dch_) on the battery’s aging characteristics is depicted in Fig. 2. For calendar aging experiments, *DOD*, *C*_ch_, and *C*_dch_ were kept at zero to eliminate the influence of cycling.

**Table 1:** The RPT protocol algorithm, as shown in Fig. 3, incorporates both constant current (CC) and constant current constant voltage (CCCV) charge or discharge steps (with current *I* and voltage *U*).

Step	Parameters	Termination	Section
1 Pause	—	*t* > 180 min	—
2 Discharge CCCV	$I=1\,{\rm{C}}\,,U={U}_{\min }$	|*I*| < C/20	—
3 Pause	—	*t* > 25 min	—
4 Charge CCCV	$I=\,{\rm{C}}\,/2,U={U}_{\max }$	|*I*| < C/20	A
5 Pause	—	*t* > 25 min	A
6 Discharge CCCV	$I=1\,{\rm{C}}\,,U={U}_{\min }$	|*I*| < C/20	A
7 Pause	—	*t* > 25 min	A
8 Charge CC	*I* = C/20	$U\ge {U}_{\max }$	B
9 Discharge CC	*I* = C/20	$U\le {U}_{\min }$	B
10 HPPC*	*SOC* = 5%, 10%, . . . , 90%, 95%	—	C,D,E
10 HPPC*	*SOC* = 50%	—	D
11 Discharge CCCV	$I=1\,{\rm{C}}\,,U={U}_{\min }$	|*I*| < C/20	—
12 Charge CCCV	$I=\,{\rm{C}}\,/2,U={U}_{\max }$	$Q\ge SO{C}_{\max }\cdot \left(\frac{| {Q}_{4}| +| {Q}_{6}| }{2}\right)$	—
13 Pause	—	*t* > 120 min	—

The charge transferred during steps 4 and 6, denoted as *Q*_4_ and *Q*_6_ respectively, is used to determine the initial state of charge ($SO{C}_{\max }$) for the subsequent aging/cycling phase (step 12). For detailed information on the HPPC* protocol, refer to Table 2.

**Table 2:** Hybrid pulse power characterization (HPPC) protocol with *step_type* calculation.

Step	Parameters	Termination	step_type
10.1 Charge CC	*I* = C/2	Q ≥ *Δ*Q_SOC_	—
10.2 Pause	—	$t > 25\min$	—
10.3 for *i*_pulse_ in [1, 2, 3]:			
10.3.1 Discharge CC	$I={{\rm{I}}}_{{i}_{{\rm{pulse}}}}$	*t* > 30 s	100 ⋅ *SOC* + 10 ⋅ *i*_pulse_ + 2
10.3.2 Pause	—	*t* > 30 s	100 ⋅ *SOC* + 10 ⋅ *i*_pulse_ + 4
10.3.3 Charge CC	$I={{\rm{I}}}_{{i}_{{\rm{pulse}}}}$	Q ≥ Q_10.3.1_	—
10.3.4 Pause	—	*t* > 30 s	—
10.3.5 Charge CC	$I={{\rm{I}}}_{{i}_{{\rm{pulse}}}}$	*t* > 30 s	100 ⋅ *SOC* + 10 ⋅ *i*_pulse_ + 1
10.3.6 Pause	—	*t* > 30 s	100 ⋅ *SOC* + 10 ⋅ *i*_pulse_ + 3
10.3.7 Discharge CC	$I={{\rm{I}}}_{{i}_{{\rm{pulse}}}}$	Q ≥ Q_10.3.5_	—
10.3.8 Pause	—	$t > 9.5\min$	—

*Δ*Q_SOC_ represents the charge increment to set a specific state of charge (SOC). *i*_pulse_ denotes the pulse index, and ${{\rm{I}}}_{{i}_{{\rm{pulse}}}}={i}_{{\rm{pulse}}}\cdot C/3$ is the current applied during each pulse. The charge transferred during steps 10.3.1 (*Q*_10.3.1_) and 10.3.5 (*Q*_10.3.5_) is used to reset the state of charge after the pulse.

**Fig. 3:**
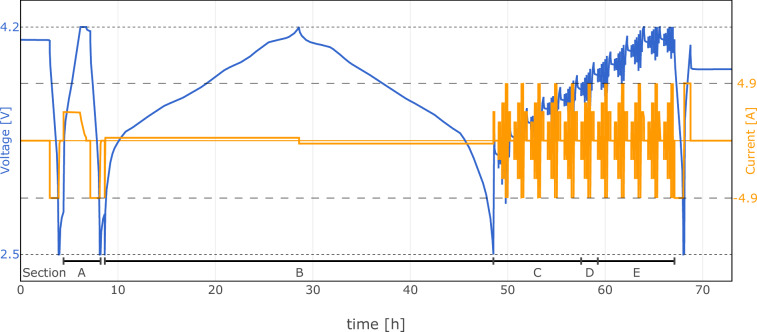
Reference performance test (RPT) protocol defined for this study. This figure depicts the RPT protocol divided into multiple sections to assess various performance metrics of lithium-ion batteries, as described in Table 1. Section A is designated for measuring the capacity of the battery. Section B provides the pseudo open circuit voltage (OCV) curves in both charge and discharge directions. Sections C, D, and E summarize the high pulse power characterization (HPPC) at different state of charge levels, with Section D specifically indicating the pulses at 50% SOC. The graph shows the current and voltage profile over time across the different sections of the RPT.

To quantify key performance metrics such as capacity and direct current internal resistance (DCIR), reference performance tests (RPTs) were conducted regularly. Three types of RPTs were employed to minimize their impact on cell degradation:

1. **Initial Check-Up (“Eingangstest” - ET) / Final Check-Up (“Ausgangstest” - AT)**: Comprehensive tests conducted at the beginning and at the end of the aging study, including measurements of capacity, pseudo open circuit voltage (pOCV) curves at C/20, and hybrid pulse power characterization (HPPC) at various SOC levels, all at three different ambient temperatures. This protocol executes all tests contained in any RPT, and is described in Table 1 along its visualization in Fig. 3 for one temperature.
2. **Extended Check-Up (exCU)**: Detailed RPT similar to ET/AT without measuring the pOCV curve. It includes a capacity measurement and HPPC tests at multiple SOCs at the reference temperature *T*_amb_ = 23 °C.
3. **Check-Up (CU)**: Shortest RPT, focusing on measuring the capacity and performing HPPC at 50% SOC.

**Fig. 4:**
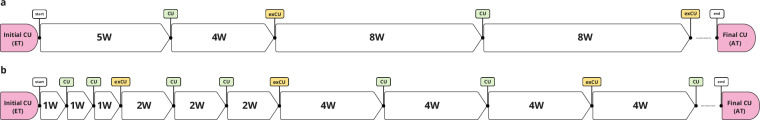
Time frame of aging intervals and regular RPT procedures. This figure illustrates the time frame for aging intervals and regular reference performance test (RPT) procedures of varying extents, including Initial tests (ET), Final tests (AT), extended check-ups (exCU), and check-ups (CU) over time. (**a**) represents the calendar aging study, while (**b**) depicts the cycle aging study. The intervals are marked in weeks (W), showing the schedule of CU and exCU tests throughout the aging process. The scope of each RPT procedure is summarized in Table 3.

The aging intervals and the sequence of RPTs are illustrated in Fig. 4, while the specific tests conducted in each RPT type are summarized in Table 3. Before the ET, three full-cycles were conducted at 23 °C to break-in the cells.

**Table 3:** Overview of test conditions, characterization sections and durations for different RPT protocols.

RPT	T_amb_ [°C]	Capacity	OCV	HPPC			approx.
Protocol				5,10,20,30,40%	50%	60,70,80,90,95%	Duration
Section		A	B	C	D	E	
ET (Init)	10,45,23	x	x	x	x	x	220h
CU	23	x			x		15h
exCU	23	x		x	x	x	30h
AT (Final)	10,45,23	x	x	x	x	x	200h

The ambient temperature (*T*_amb_) settings, measured test sections (Capacity, OCV, HPPC at various SOC levels), and the approximate duration for each protocol are provided.

### Design of Experiments (DoE)

The aging study was conducted in two consecutive stages within a multi-stage experimental design^87^. The design of experiments for the first stage is based on the assumption of no a priori knowledge about the aging behavior of the cells. The DoE for stage 2 is based on stage 1 results. Both stages were planned to be carried out for approximately one year each.

Ambient temperatures were constrained to 10, 23, 35 and 45 °C due to the limited availability of thermal chambers, where the above room temperature conditions 23, 35 and 45 °C were used during cycling to capture realistic and application-motivated scenarios for stationary battery energy storage systems (BESS)^88^. By focusing on these temperatures, our study provides a valuable dataset that reflects common operational conditions, ensuring its relevance and applicability to a wide array of practical applications. Current rates for the experiments were limited by the manufacturer’s data sheet to *C*_ch_ = [0.05 ⋯ 1] and *C*_dch_ = [0.05 ⋯ 2]^86^. $SO{C}_{\max }$ and *DOD* were restricted to maintain the state of charge within 20−80% during cycling to avoid constant voltage (CV) scenarios. This range was chosen based on pre-tests indicating that limiting the *SOC* within these bounds allows for a constant current (CC) to be maintained during cycling. By ensuring CC charging and discharging profiles throughout the study, variations in the aging profile that could arise from declining currents resulting in significant increase of (dis)charging duration during CV, phases were aimed to be avoided.

For calendar aging, cells were stored at open-loop conditions with *SOC* ranging from 5% to 100%, using CCCV charging to set $SO{C}_{\max }$.

#### Stage 1

The objective of the first stage was to gather preliminary aging knowledge about the aging behavior of the battery cells. A full factorial (FF) motivated design was used for the calendar aging study to define the aging conditions of 11 test points. Given the high number of degrees of freedom and the resulting extensive combination of test conditions, a conditioned Latin hypercube (cLH) sampling strategy was chosen for the cycle aging study to efficiently allocate 25 test points to the available equipment^89^.

#### Stage 2

A new batch of battery cells was tested during stage 2. The upper $SO{C}_{\max }$ limit was restricted to 80% for stage 2 of the calendar aging study as non-monotonic behavior was observed above 80% during stage 1, which is not captured by the model.

Stage 2 of our experimental design was divided into two sub-studies, one of which is informed by the insights gained from stage 1. The second sub-study uses stage 1 methodology where no pre-assumtions are necessary. These are described in more detail below:

1. **Optimized Experimental Design (pi-OED):** The first sub-study in stage 2 focused on optimizing the experimental design to refine parameter estimation for a given semi-empirical aging model (see SI S.1.1), which is motivated by the aging model described by Muehlbauer *et al*.^90^. This model describes both calendar and cycle aging aspects by independent parametric sub-models (SI equations (S.2) and (S.3)), respectively. Using the methodology of parameter individual optimal experimental design (pi-OED)^91^, we aimed to enhance the accuracy of temperature-related parameters in the calendar aging model and current rate-related parameters in the cycle aging model. Specifically, this sub-study included:
  **Calendar Aging:** Four test points were selected to improve the estimation of temperature-related parameters (${\theta }_{0}^{{\rm{cal}}}$).
  **Cycle Aging:** Twelve test points were chosen to refine the estimation of current rate-related parameters (${\theta }_{2}^{{\rm{cyc}}}$ and ${\theta }_{3}^{{\rm{cyc}}}$).

The selection of these test points was based on their potential to improve the precision of parameter estimation using Fisher Information-based pi-OED methodology.

2. **Exploratory Study Without Pre-assumptions:** The second sub-study in Stage 2 was planned similarly to Stage 1, without any pre-assumptions. This approach ensured a broad exploration of battery aging behaviors under various conditions, serving as a reference for quantifying the efficiency increase of optimized experimental design. This study included:
  **Calendar Aging:** Three test points were manually chosen to fill the grid of the full factorial sampling matrix and four additional test points to further explore the design space.
  **Cycle Aging:** Twelve test points were planned using a conditioned Latin hypercube (cLH) sampling strategy, allowing for a wide-ranging investigation of the experimental design space.

**Fig. 5:**
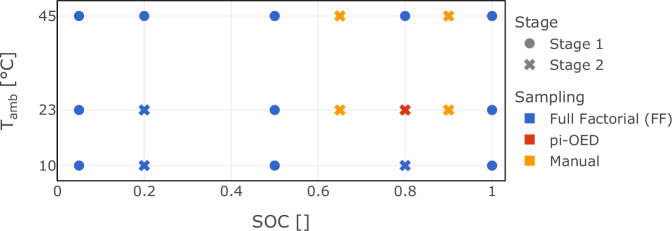
Design of Experiments for multi-stage Li-ion battery calendar aging characterization. This figure depicts the design of experiments (DoE) for the two stages of the multi-stage experimental study aimed at characterizing Li-ion battery calendar aging. The symbols represent different stages, with stage 1 and stage 2 marked accordingly. The colors indicate the methodology used to generate or compute the given DoE, including Full Factorial (FF), pi-OED, and manual selection. The graph plots the state of charge (*SOC*) against ambient temperature (*T*_amb_) in degrees Celsius (°C) for each stage, illustrating the test points (TP) under which the aging characterization was performed.

**Fig. 6:**
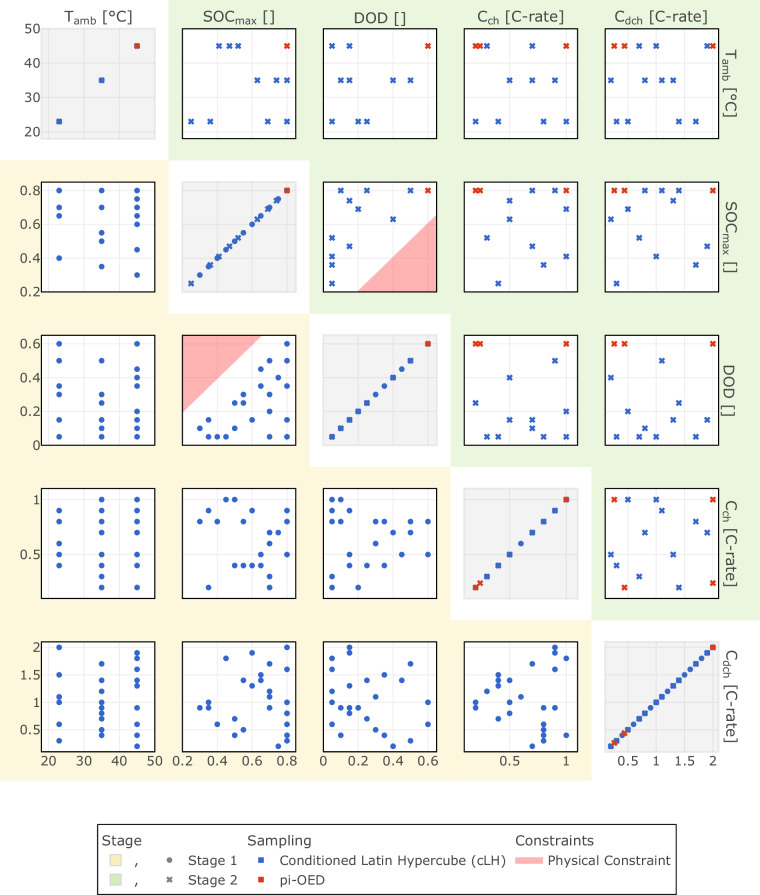
Design of Experiments for multi-stage Li-ion battery cycle aging characterization. This figure presents the DoE for the two stages of the multi-stage experimental study focused on Li-ion battery cycle aging. The different stages are represented by specific symbols, with stage 1 highlighted in yellow in the lower left part and stage 2 highlighted in green in the upper right part. The methodologies used to generate or compute the DoE are indicated by different colors, including Conditioned Latin Hypercube (cLH) sampling and pi-OED, along with physical constraints. The diagonal line combines all test points from both stages. The graphs display the relationships between key experimental variables: ambient temperature (*T*_amb_) in degrees Celsius (°C), maximum state of charge ($SO{C}_{\max }$), depth of discharge (*DOD*), charge and discharge current (*C*_ch_, *C*_dch_). These plots illustrate the test points and constraints applied during this cycle aging characterization.

The designs of experiments for both stages are illustrated in Fig. 5 for the calendar aging study and Fig. 6 for the cycle aging study. In Fig. 6, the lower left section (yellow background) represents the first stage, while the upper right section (green background) shows the second stage. The diagonal plots display the distribution of test points for the whole cycle aging dataset.

## Data Records

All data files are available under a CC-BY-4.0 licence at figshare^92^.

Before starting the aging study, each cell was characterized using a standard entry protocol. This protocol involved measuring its weight and 1kHz-resistance at room temperature (controlled at 23 °C). The results were documented in the *experiments_meta.csv* file for each cell, along with information about the used measurement equipment, and the aging condition of every tested cell, as summarized in Table 4. We categorize the dataset into reference performance tests (RPT) and cycling measurements (“Zyklen” - ZYK). The RPT files encompass the initial check-up (“Eingangstest” - ET), the extended check-up (exCU), the standard check-up (CU), and the final check-up (“Ausgangstest” - AT). ZYK files contain the data recorded during cycling. Note that there are no files for calendar aging storage measurements, as these measurements were conducted in an open-circuit condition. All files are provided in comma-separated values (CSV) format and contain time series data for the measured values as provided in Table 5. All measurement files include a *step_type* column to simplify extraction of specific test sections. Table 6 summarizes all *step_types* used throughout the study, with allocation to RPT steps and calculation details during HPPC tests given in Tables 1 and  2, respectively.

**Table 4:** Header description of the *experiments_meta.csv* overview file.

Value	Unit	Description
serial_internal	—	Internally used cell identifier
serial	—	Unique cell identifier per stage
lab	—	Laboratory in which the cell was tested
type	—	Calendar (k) or cycle (z) aging characterization
tp	—	Unique test point id per stage
cell	—	Unique cell id per test point
amb_temp_tp	°C	Ambient temperature controlled by climate chamber during aging phases
soc_max_tp	—	State-of-charge setpoint after RPTs
dod_tp	—	Depth-of-discharge for cycling protocol
c_ch_tp	C-rate	Charge C-rate for cycling protocol
c_dch_tp	C-rate	Discharge C-rate for cycling protocol
sampling	—	Method of DoE sampling, i.e. FF, cLH, pi-OED or manual
stage	—	Unique stage identifier
m_0	gram	Weight of the cell after delivery
scale	—	Measuring equipment used to weigh the cell
R_1khz_0	*Ω*	1kHz-resistance of the cell after delivery at room temperatue (approx. 23°C)
resistance_meter	—	Measuring equipment used to measure the 1kHz resistance of the cell
U_0	V	Voltage of the cell after delivery
volt_meter	—	Measuring equipment used to measure the voltage of the cell

**Table 5:** Header description of RPT and cycling measurement files.

Value	Unit	Description
run_time	HH:mm:ss.SSS	Time since the start of the measurement
c_vol	V	Cell voltage measured with 4-wire measurement
c_cur	A	Cell current, negative during discharging
c_surf_temp	°C	Cell surface temperature, measured on the cylinder mantle
amb_temp	°C	Ambient temperature, representing the climate chamber temperature
step_type	—	Test section code as described in Table 6

**Table 6:** Decoding of *step_type* variable in data records.

Value	Section	Description
21	A	Capacity measurement, CCCV charging (CC at C/2, with CV at 4.2V and C/20 cut off)
22	A	Capacity measurement, CCCV discharging (CC at 1C, with CV at 2.5V and C/20 cut off)
31	B	Open circuit voltage curve measurement, CC charging (C/20)
32	B	Open circuit voltage curve measurement, CC discharging (C/20)
511, …, 9534	C,D,E	HPPC pulses, see Table 2 for detailed calculation
5011, …, 5034	D	HPPC pulses at 50% SOC, see Table 2 for detailed calculation
41	—	Cycling, charge phase
42	—	Cycling, discharge phase

Each data file is accompanied by a *_meta.txt file, which describes the measurement setup for the given measurement, including the laboratory identifier, the internal cell serial, test point identifier, measurement start date, the name of the used measurement device, the test channel, the name of the used climate chamber, its temperature setpoint and information about the position of the cell inside the climate chamber.

The dataset is divided according to the two defined stages into *Stage_1* and *Stage_2*. Each stage consists of multiple test points (TP), that represent the corresponding test conditions for calendar (“kalendarisch” - k) and cycle (“zyklisch” - z) aging, also referred to as *aging type*. Every TP is repeated with three cells (01-03) following the described testing protocol (see Methods). For all calendar aging TPs, another three cells (04-06) are tested with only performing an initial ET and a final AT. Instead of regular RPTs during aging, the cells were recharged to their reference open circuit voltage (OCV) using CCCV charging to reset their storage *SOC*. However, no characterization was performed. The reference OCV was measured at the end of the ET, after setting the $SO{C}_{\max }$ according to the corresponding test point. The RPT data and cycling data (only for cycle aging cells) for every tested cell are stored in a corresponding folder.

## Technical Validation

### Validation of comparability among all cells

Although cells originate from the same manufacturer and are of the same type, variations in production time may lead to variations in electrode characteristics e.g. due to slight changes in cell material composition. The open circuit voltage (OCV) curve of a cell, more specifically its derivative with respect to capacity known as differential voltage analysis (DVA) curve unfolds insights into the electrode material composition of the cell^93^.

Therefore, comparing the DVA curves during the initial RPT of each individual cell with the arithmetic mean DVA curve of all cells provides information on whether it can be assumed that the cells behave equally and thus can be compared. To evaluate the assumption of the mean as a surrogate for each cells DVA, the coefficient of determination (*R*^2^) is calculated for each cell individually. For all cells, the coefficient of determination *R*^2^ > 0.99 indicates that the cells belong to the same statistical ensemble with a significance level of *α* = 0.01, and thus can be treated as originating from a single batch.

### Validation of capacity and resistance degradation trends

**Fig. 7:**
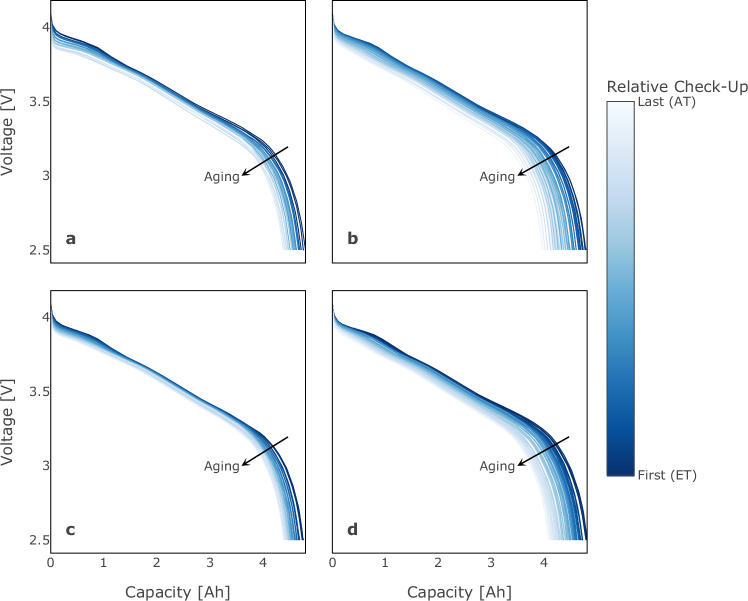
Voltage discharge curves vs 1 C discharge capacity. This figure illustrates the voltage discharge curves as a function of discharge capacity with a 1 C discharge current. The curves are colored according to the relative RPT for all cells at exemplary selected test points. (**a**) and (**b**) show the results for test points TP_k11 and TP_z17 in stage 1, respectively. (**c**) displays the results for test point TP_k11, while (**d**) presents the results for TP_z16 in stage 2.

**Fig. 8:**
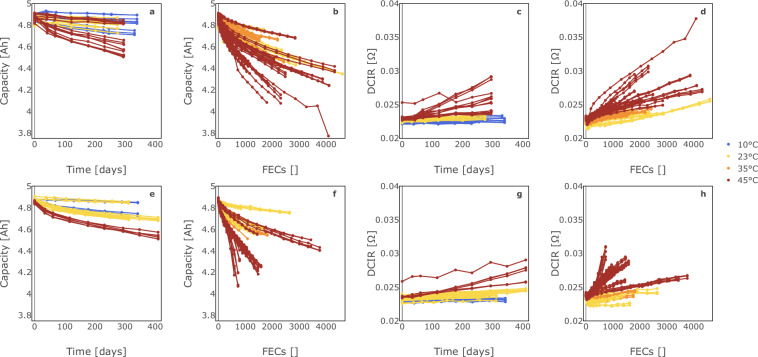
Overview of all aging trends during the two-stage calendar and cycle aging study. This figure shows the aging trajectories of all tested cells including potential outliers, where the first row corresponds to stage 1, while the second row shows the results of stage 2. The left four graphs illustrate the capacity loss over time and fully equivalent cycles (FEC) for calendar and cycle aging, respectively. The right four graphs show the corresponding increase in DC internal resistance (DCIR). (**a**), (**c**), (**e**), and (**g**) present data for calendar aging over time in days, whereas (**b**), (**d**), (**f**), and (**h**) display results for cycle aging vs FECs.

**Fig. 9:**
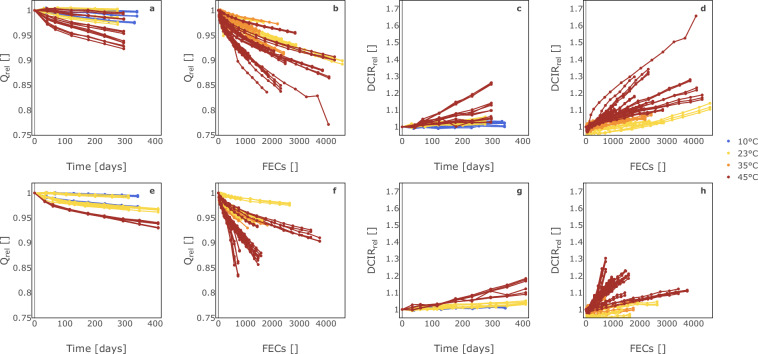
Overview of all relative aging trends during the two-stage calendar and cycle aging study. This figure shows the aging trajectories of all tested cells including potential outliers, where the first row corresponds to stage 1, while the second row shows the results of stage 2. The left four graphs illustrate the relative capacity (Q_rel_) over time and fully equivalent cycles (FEC) for calendar and cycle aging, respectively. The right four graphs show the corresponding relative increase in DC internal resistance (DCIR_rel_). (**a**), (**c**), (**e**), and (**g**) present data for calendar aging over time in days, whereas (**b**), (**d**), (**f**), and (**h**) display results for cycle aging vs FECs.

Degradation mechanisms during aging of lithium ion batteries lead to capacity loss and resistance growth^94^, both of which influence the trajectories of a voltage discharge curve vs capacity. The voltage discharge curves indicated in Fig. 7 show the capacity loss during constant current discharge (with a discharge current of 1 C) by increasing compression along the capacity axis over degradation. Increasing inner resistance leads to higher overpotential during constant current discharge, which becomes apparent when the voltage drops. Capacity was determined during regular RPTs as the mean of charge and discharge capacity (see Methods for protocol). The DC internal resistance (DCIR) was determined using a discharge pulse with a current of 1C at a state of charge of 50%. The DCIR can be extracted by taking the voltage difference induced by the pulse current after 10 seconds and divide it by the applied current (1 C). Figure 8 shows the capacity loss and DCIR growth during stage 1 for calendar (a, c) and cycle (b, d) aging, and during stage two for calendar (e, g) and cycle (f, h) aging, respectively. Figure 9 shows the respecive relative capacity loss Q_rel_ = Q/Q_0_ and relative DCIR increase DCIR_rel_ = DCIR/DCIR_0_, which can provide a clearer comparison of aging trends. The degradation trends follow the expected behavior with sublinear and some superlinear and knee-shaped trends for capacity decay and mainly linear trends for DCIR increase.

### Known incidents encountered during the study

A number of incidents occurred during the aging study. These potential outliers should be handled prior further processing, unless outliers are explicitly wanted for non-standard analytics e.g. with respect to test robustness of algorithms.

#### Stage 1

- **TP_k[08,09,10,11]_[01,04]_01_ET_T23:** Discharge during capacity cycle without constant voltage (CV) phase.
  → Repeated capacity measurement after OCV phase and replaced the failed capacity cycle. (Charge OCV may be corrupted).
- **TP_k11_02_03_exCU:** Tester malfunction.  → Charged cell from 80% to 100% $SO{C}_{\max }$ manually.
- **TP_z25_01_01_ET_T45:** Cell incorrectly placed in cell holder, undefined load.
- **TP_z24_01_02_ZYK:** Cell incorrectly placed in cell holder, undefined load during cycling.
- **TP_z19_01_04_ZYK:** Tester malfunction. Cycling data missing. Cycling itself was performed correctly.
- **TP_z25_01_04_ZYK:** Cell incorrectly placed in cell holder, undefined load during cycling.
- TP_z[04-15]_*_04_ZYK: Lab blackout.  → Cycling continued after $SO{C}_{\max }$ reset.
- **TP_z01_03_11_CU:** Tester malfunction.  → Concatenated files at start of charge CV phase during capacity measurement.
- **TP_z17_03_13_exCU:** Tester malfunction. Incorrect $SO{C}_{\max }$.
- TP_z[16-25]_*_16_ZYK: Lab blackout.  → Cycling continued after $SO{C}_{\max }$ reset.
- **TP_z20_03_16_ZYK:** Tester malfunction. $SO{C}_{\max }$ reset (see above) failed. Cycling continued at wrong $SO{C}_{\max }$.
- **TP_z17_03_17_CU:** Tester malfunction. Incorrect $SO{C}_{\max }$.  → *U*_*lb*_ corrected during cycling in *TP_z17_03_18_ZYK* according to twin cells.
- **TP_z17_02_19_exCU:** Tester malfunction.  → Cell charged to $SO{C}_{\max }$ manually.
- **TP_z21_01_22_ZYK:** Tester malfunction. Cycling data partially missing. Cycling itself was performed correctly.
- **TP_z24_01_22_ZYK:** Tester malfunction. Cycling data partially missing. Cycling itself was performed correctly.
- **TP_z25_02_22_ZYK:** Tester malfunction. Cycling data partially missing. Cycling itself was performed correctly.

#### Stage 2

- TP_z[13-17,19,20,22,23,24]_*_14_ZYK: Climate chamber downtime. No measurements recorded. No cycling was performed as tests were paused for 15 days.
- **TP_z07_03_16_ZYK:** Cell holder connection failures. undefined load during cycling.
- TP_z[12,18,21]_*_22_ZYK: Lab downtime for 4 days, while cycling was paused.  → Cycling was continued after the downtime with a 3 hours tempering pause.
- TP_z[13-17,19,20,22,23,24]_*_22_ZYK: Climate chamber downtime for 20 days, while cycling was paused.  → Cycling was continued after the downtime with a 3 hours tempering pause.
- TP_z[01,03]_*_24_ZYK: Tester malfunction. Cycling data missing. Cycling itself was performed correctly for 33 days.
- TP_z[04-11]_*_24_ZYK: Tester malfunction. 2 days of cycling data missing. Cycling itself was performed correctly.
- TP_z12_*_31_AT_T45: Tester malfunction. Test aborted during discharge of the capacity cycle. Continued after 55 minutes.

Furthermore, for all cycle and calendar aging tests the indicators extracted from the final RPT (AT) at 23 °C may deviate from the expected degradation trajectory due to the previously conducted RPTs at 10 °C and 45 °C, which caused further cycling stress to the cells. The authors kindly ask all researchers using this dataset to provide information about possible further irregularities they may have discovered.

## Usage Notes

During the study 279 individual battery cells were aged under 93 different conditions for about one year each, resulting in an accumulated aging time of almost 250 years. The dataset takes nearly 10 gigabytes (GB) compressed as provided on the figshare repository^92^, expanding to approximately 100GB uncompressed.

Individual technically corrupted files and potentially consecutive files should be dropped from the dataset prior its processing, unless outlier behavior is intended to be analyzed (see Technical Validation for known issues during the study).

Exemplary Python processing functions along with a Jupyter Notebook to explain its usage are provided (see Code availability below).

## Supplementary information

Supplementary Information

## Data Availability

No custom code was used to generate the dataset. Python (3.11) and plotly (5.22) were used to concatenate, process and visualize data. Custom scripts and a Jupyter Notebook describing basic usage of the dataset is available at https://github.com/fst2112/Multi-Stage-Lithium-Ion-Battery-Aging-Dataset-Analysis. Please contact the corresponding authors for further code requests.
