# Supplementary material for: A multi-stage lithium-ion battery aging dataset using various experimental design methodologies
Source: Sci Data. 2024 Sep 19;11:1020. doi: 10.1038/s41597-024-03859-z (PMC11412976; doi:10.1038/s41597-024-03859-z)
Supplement: Supplementary file 1 — Supplementary Information [file 41597_2024_3859_MOESM1_ESM.pdf]

A multi-stage lithium-ion battery aging dataset using various experimental design methodologies

Florian Stroebl[1], Ronny Petersohn[2], Barbara Schricker[3], Florian Schaeufl[1], Oliver Bohlen[1,*], and Herbert Palm[1,*]

[1]Institute for Sustainable Energy Systems (ISES), Munich University of Applied Sciences, Munich, 80335, Germany
[2]Hoppecke Systemtechnik GmbH, Advance Development, Zwickau, 08056, Germany
[3]Siemens AG, Technology, Erlangen, 91058, Germany
[*]Corresponding Author(s): H. Palm (palm@hm.edu), O. Bohlen (bohlen@hm.edu)

S.1 Supplemental

S.1.1 Semi-empirical aging models

Motivated by Muehlbauer et al.[90], the total relative capacity loss of a lithium ion battery cell $Q_{\text{loss,rel}}$ is assumed to result from a linear superposition of independent calendar aging $Q^{cal}_{\text{loss,rel}}$ and cycle aging $Q^{cyc}_{\text{loss,rel}}$ related losses according

$$Q_{\text{loss,rel}} = Q^{cal}_{\text{loss,rel}} + Q^{cyc}_{\text{loss,rel}}\,, \tag{S.1}$$

where the calendar aging aspect of the model can be described by

$$Q^{\text{cal}}_{\text{loss,rel}}(T, SOC, t; \boldsymbol{\theta}_{\text{cal}}) = (1 - EOL_C) \cdot \left(e^{-\theta_0^{\text{cal}} \cdot \left(\frac{1}{T} - \frac{1}{T_{\text{ref}}}\right)}\right) \cdot \left(\frac{SOC}{SOC_{\text{ref}}}\right)^{\frac{1}{\theta_1^{\text{cal}}}} \cdot \left(\frac{t}{t_{\text{ref}}}\right)^{\theta_2^{\text{cal}}}\,, \tag{S.2}$$

and the cycle aging aspect by

$$Q^{\text{cyc}}_{\text{loss,rel}}(T, SOC_{\text{max}}, C_{\text{ch}}, C_{\text{dch}}, DOD, FEC; \boldsymbol{\theta}_{\text{cyc}}) = \tag{S.3}$$
$$(1 - EOL_C) \cdot \left(e^{-\theta_0^{\text{cyc}} * \left(\frac{1}{T} - \frac{1}{T_{\text{ref}}}\right)}\right) \cdot \left(\frac{SOC_{\text{max}}}{SOC_{\text{max,ref}}}\right)^{\frac{1}{\theta_1^{\text{cyc}}}} \cdot \left(\frac{C_{\text{ch}}}{C_{\text{ch,ref}}}\right)^{\frac{1}{\theta_2^{\text{cyc}}}} \cdot \left(\frac{C_{\text{dch}}}{C_{\text{dch,ref}}}\right)^{\frac{1}{\theta_3^{\text{cyc}}}} \cdot \left(\frac{DOD}{DOD_{\text{ref}}}\right)^{\frac{1}{\theta_4^{\text{cyc}}}} \cdot \left(\frac{FEC}{FEC_{\text{ref}}}\right)^{\theta_5^{\text{cyc}}}.$$

Both aging submodels are describing the relative capacity loss $Q_{\text{loss,rel}}$ above a capacity end-of-life criterion EOL_C, where each aging factor is scaled by a reference value and associated to one parameter of a parameter vector $\boldsymbol{\theta}$. The calendar aging model depicts the $Q^{cal}_{loss,rel}$ dependence on ambient storage temperature T and storage state of charge (SOC) during an open-loop storage condition for t days by the parameter vector $\boldsymbol{\theta}_{\text{cal}} = (\theta_0^{\text{cal}}, \theta_1^{\text{cal}}, \theta_2^{\text{cal}})$. The cycle aging model describes the $Q^{cyc}_{loss,rel}$ dependence on ambient temperature T, maximum state of charge during cycling SOC_{max}, the charge and discharge C-rates C_{ch} and C_{dch} with a cycle depth of discharge DOD along FEC fully equivalent cycles on capacity loss during cycling by its corresponding parameter vector $\boldsymbol{\theta}_{\text{cyc}} = (\theta_0^{\text{cyc}}, \cdots, \theta_5^{\text{cyc}})$. The relative capacity extracted from the i-th reference performance

test (RPT) is always related to the initial capacity Q_0 measured during the initial RPT ("Eingangstest" - ET):

$$Q_{\mathrm{rel},i} = \frac{Q_i}{Q_0} \tag{S.4}$$

References

90. Muehlbauer, M., Rang, F., Palm, H., Bohlen, O. & Danzer, M. A. Pareto-optimal power flow control in heterogeneous battery energy storage systems. *J. Energy Storage* **48**, 103803, https://doi.org/10.1016/j.est.2021.103803 (2022).
